# Pilomatrixoma of the Ankle: A Case Report

**DOI:** 10.7759/cureus.64238

**Published:** 2024-07-10

**Authors:** Sayed Mohamed S Ahmed

**Affiliations:** 1 College of Medicine, I.M. Sechenov First Moscow State Medical University, Moscow, RUS

**Keywords:** magnetic resonance imaging (mri), fine-needle aspiration biopsy, surgical excision, histopathology, differential diagnosis, subcutaneous nodule, hair follicle matrix, benign skin tumor, calcifying epithelioma, pilomatrixoma

## Abstract

Pilomatrixoma, also known as calcifying epithelioma of Malherbe, is a rare benign skin tumor originating from hair follicle matrix cells. It typically presents as a firm, painless subcutaneous nodule, most commonly found in the head, neck, and upper extremities. Pilomatrixoma can occasionally appear in atypical locations, posing a diagnostic challenge due to its nonspecific clinical presentation. A 43-year-old female presented with a painless, slowly enlarging mass on the lateral side of her left ankle, which had been present for approximately one year. Physical examination revealed a firm, well-circumscribed subcutaneous nodule measuring about 2 cm in diameter with normal overlying skin. An MRI of the left ankle demonstrated a well-circumscribed, subcutaneous mass with heterogeneous signal intensity, consistent with calcifications, suggesting pilomatrixoma. A fine-needle aspiration biopsy confirmed the presence of basaloid cells, shadow cells, and areas of calcification. The lesion was surgically excised, and histopathological examination validated the diagnosis of pilomatrixoma. The patient had an uneventful postoperative course, with no recurrence at the six-month follow-up. This case underscores the importance of considering pilomatrixoma in the differential diagnosis of subcutaneous nodules, even in unusual locations. A comprehensive diagnostic approach, including clinical evaluation, imaging, and histopathological examination, is essential for an accurate diagnosis. Surgical excision with clear margins is the treatment of choice, ensuring low recurrence rates and excellent patient outcomes. This report enhances the understanding of pilomatrixoma and highlights the necessity for a multimodal diagnostic strategy in managing this rare condition effectively.

## Introduction

Pilomatrixoma, also known as calcifying epithelioma of Malherbe, is a benign skin tumor originating from the hair follicle matrix cells. First described by Malherbe and Chenantais in 1880, pilomatrixoma typically presents as a solitary, firm, subcutaneous nodule that is most commonly found in the head, neck, and upper extremities [1,2]. Although it can occur at any age, pilomatrixoma is predominantly seen in children and young adults, with a slight female predominance. The lesion is usually asymptomatic and slow-growing, making it easy to overlook or misdiagnose [2,3].

The pathogenesis of pilomatrixoma involves the abnormal differentiation of hair matrix cells, leading to the formation of basaloid cells, shadow or ghost cells, and areas of calcification. Clinically, pilomatrixomas can mimic other benign and malignant skin conditions, such as epidermal cysts, lipomas, and even sarcomas, making an accurate diagnosis challenging [1-3]. Diagnosis is often supported by imaging studies, such as ultrasound, which typically reveal a well-defined, hypoechoic mass with or without calcifications. However, a definitive diagnosis requires histopathological examination, demonstrating the characteristic cellular components [3,4]. Management of pilomatrixoma involves complete surgical excision, which is generally curative with a low recurrence rate [3].

## Case presentation

A 43-year-old female patient presented to the outpatient dermatology clinic with a chief complaint of a painless, slowly enlarging mass on her left ankle, located laterally. The patient reported that the lesion had been present for approximately one year and had gradually increased in size. There was no history of trauma, infection, or previous similar lesions. The patient’s medical history was significant for well-controlled hypertension and hypothyroidism, for which she was taking amlodipine and levothyroxine, respectively. She had no known allergies and denied any family history of similar skin lesions or malignancies.

On physical examination, a firm, well-circumscribed, subcutaneous nodule measuring approximately 2 cm in diameter was palpated over the lateral aspect of the left ankle. The overlying skin appeared normal without any signs of erythema, ulceration, or hyperpigmentation. There was no associated tenderness, warmth, or regional lymphadenopathy. The rest of the physical examination was unremarkable, and no other similar lesions were identified elsewhere on her body.

Given the patient’s clinical presentation, a differential diagnosis was considered, including benign entities such as epidermal inclusion cyst, lipoma, ganglion cyst, and more rare conditions like dermatofibroma or pilomatrixoma. Malignant conditions like soft tissue sarcoma or metastatic disease were also considered, although deemed less likely given the lesion’s characteristics and the patient’s overall health status.

**Figure 1:**
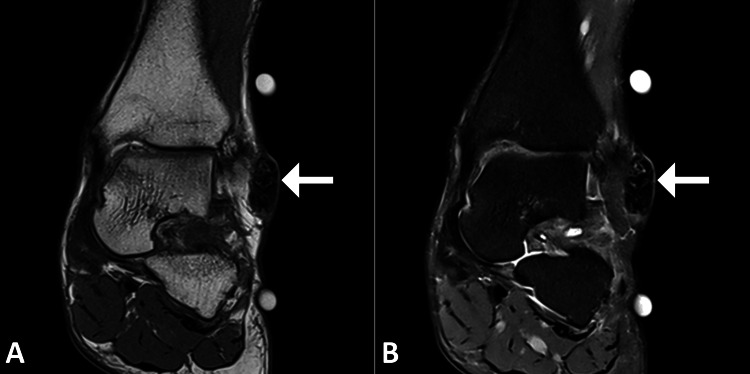
MRI images of the left ankle Coronal images of the ankle in PD-weighted (A) and T2-fat-saturated (B) images show an ovoid lesion over the lateral aspect of the ankle with heterogeneous signal intensity (arrow). MRI: magnetic resonance imaging

To further evaluate the nature of the lesion, an ultrasound of the left ankle was performed, revealing a well-defined, hypoechoic mass within the subcutaneous tissue. No internal vascularity was noted on Doppler imaging, supporting the suspicion of a benign process. Subsequently, an MRI of the left ankle was obtained to provide more detailed imaging. The MRI demonstrated a well-circumscribed, subcutaneous mass with heterogeneous signal intensity, consistent with calcifications and characteristic of pilomatrixoma (Figure 1).

To confirm the diagnosis and rule out malignancy, a fine-needle aspiration (FNA) biopsy was conducted. The cytology report from the FNA showed clusters of basaloid cells, shadow cells, and areas of calcification, which are characteristic findings suggestive of pilomatrixoma.

Given these findings, an excisional biopsy was recommended for a definitive diagnosis and treatment. The patient underwent surgical excision of the lesion under local anesthesia. The excised specimen was sent for histopathological examination, which confirmed the diagnosis of pilomatrixoma, characterized by the presence of basaloid cells, ghost cells, and calcifications. The surgical margins were clear of tumor cells, indicating complete excision.

The patient’s postoperative course was uneventful, with no complications reported. She was advised on wound care and scheduled for a follow-up visit two weeks after surgery. At the follow-up appointment, the surgical site was well-healed with no signs of infection or recurrence of the lesion. The patient reported no pain or discomfort and expressed satisfaction with the cosmetic outcome.

Further follow-up at six months post-surgery revealed no evidence of recurrence, and the patient remained asymptomatic. She was educated about the benign nature of pilomatrixoma but was advised to monitor for any new or recurrent lesions and to report any changes promptly.

## Discussion

The presented case of pilomatrixoma of the left ankle highlights several key aspects of this rare and benign cutaneous neoplasm, contributing valuable insights to the existing literature [1-4]. Pilomatrixoma, although predominantly occurring in the head, neck, and upper extremities, can occasionally present in atypical locations, such as the lower extremities, as demonstrated in this case.

One of the critical points in the discussion of pilomatrixoma is its pathogenesis and histopathological characteristics. Pilomatrixomas arise from hair follicle matrix cells and are histologically characterized by the presence of basaloid cells, ghost or shadow cells, and calcifications [2,3]. The basaloid cells are small, deeply basophilic cells with scant cytoplasm, while the ghost cells are anucleate cells that retain the shape of the original cells but are filled with keratin. The calcification within the lesion is a hallmark feature and aids in differentiation from other benign and malignant entities. These histopathological features were clearly evident in the excised specimen from our patient, reinforcing the diagnosis of pilomatrixoma [2-4].

Clinically, pilomatrixomas are often misdiagnosed due to their nonspecific presentation. Common misdiagnoses include epidermal inclusion cysts, lipomas, ganglion cysts, and dermatofibromas. This case further emphasizes the necessity of considering pilomatrixoma in the differential diagnosis of firm, painless subcutaneous nodules [1,5]. The use of ultrasound as an initial imaging modality can be particularly beneficial. In our case, the ultrasound findings of a well-defined hypoechoic mass with no internal vascularity were consistent with a benign process, steering the diagnostic considerations toward pilomatrixoma [4-6].

Surgical excision is the treatment of choice for pilomatrixoma, aiming for complete removal to minimize the risk of recurrence. In our patient, the lesion was excised with clear margins, and the postoperative course was uneventful. The clear margins are crucial in reducing the likelihood of recurrence, which, although rare, can occur if the lesion is not completely excised [1,6]. This reinforces the importance of thorough surgical techniques and follow-up [4].

The recurrence rate of pilomatrixoma is generally low, estimated at around 2-3%, but it necessitates regular follow-up to ensure there are no signs of recurrence [3-5]. Our patient was monitored postoperatively and showed no evidence of recurrence at the six-month follow-up, which aligns with the expected outcomes of well-excised pilomatrixomas.

## Conclusions

This case of pilomatrixoma of the left ankle emphasizes the importance of considering this rare benign neoplasm in the differential diagnosis of subcutaneous nodules, even in atypical locations. The case highlights the critical role of a comprehensive diagnostic approach involving clinical evaluation, imaging, and histopathological examination to achieve an accurate diagnosis. Surgical excision remains the definitive treatment, with a low recurrence rate when complete removal is achieved. This report contributes to the broader understanding of pilomatrixoma and reinforces the necessity for clinicians to maintain a high index of suspicion and utilize a multimodal diagnostic strategy for effective management and optimal patient outcomes.
